# A decade of tobacco control efforts: Implications for tobacco smoking prevalence in Eastern Mediterranean countries

**DOI:** 10.1371/journal.pone.0297045

**Published:** 2024-02-23

**Authors:** Negar Taheri, Pedram Fattahi, Elnaz Saeedi, Maryam Sayyari, Sepideh Abdi, Mina Khaki, Navid Rahimi, Rouhollah K. Motamedi, Fereshte Lotfi, Mojtaba Vand Rajabpour, Saeed Nemati

**Affiliations:** 1 Cancer Research Center, Cancer Institute, Tehran University of Medical Sciences, Tehran, Iran; 2 Student research Center, Qazvin University of Medical Sciences, Qazvin, Iran; 3 School of Medicine, Tehran University of Medical Sciences, Tehran, Iran; 4 Department of Physical Medicine and Rehabilitation, School of Medicine, Shahid Beheshti University of Medical Sciences, Tehran, Iran; 5 Medical Genomic Research Center, Tehran Medical Sciences, Islamic Azad University, Tehran, Iran; 6 Department of Biostatistics and Epidemiology, School of public health, Tehran University of Medical Sciences, Tehran, Iran; Shahid Beheshti University of Medical Sciences School of Medicine, ISLAMIC REPUBLIC OF IRAN

## Abstract

**Background and objectives:**

This study aimed to assess the association between the 10-year implementation of tobacco control policies, cigarette affordability index and changes in tobacco smoking prevalence across Eastern Mediterranean (EMR) countries.

**Materials and methods:**

An ecologic study was conducted using EMR countries as the analytical unit. Data from three sources were utilized: the MPOWER scale to measure tobacco control policy implementation (2010–2020), the tobacco affordability index (expressed as a percentage of GDP per capita required to purchase 2000 cigarettes, from 2010 to 2020), and national tobacco smoking prevalence data for EMR countries (2010–2023). Linear Fixed-effect regression was employed to investigate associations between changes in MPOWER scores, the cigarette affordability index, and alterations in tobacco prevalence over a decade.

**Results:**

Statistically significant inverse associations were observed between changes in MPOWER scores and tobacco smoking prevalence among both men and women in EMR countries (P-value<0.05). Each unit increase in MPOWER score corresponded to a 0.26% reduction in tobacco prevalence among men and a 0.12% reduction among women. The regression model revealed that each unit increase in the cigarette affordability index was linked to a 0.9% decrease in tobacco smoking prevalence across EMR countries (P-value<0.05). Furthermore, even after adjusting for multiple confounders, significant inverse associations were noted between tobacco monitoring (β = -0.41), health warning (β = -0.45), and changes in tobacco smoking prevalence (P-value<0.05).

**Conclusion:**

This study underscored the effectiveness of enhancing the implementation of tobacco control policies and increasing the cigarette affordability index as preventive measures to reduce tobacco smoking prevalence in EMR countries over the past decade.

## Introduction

Tobacco is the most major independent cause of death in the world [1]. Tobacco smoking leads more than 7.5 million death globally in 2020 and it plays a critical role in the etiology of six out of eight leading causes of death like cancer, cardiovascular diseases, and chronic obstructive pulmonary diseases [2, 3]. It has also a substantial economic burden on people and health systems through imposing direct healthcare costs and reducing people and the nation’s productivity. The estimated annual global costs associated with tobacco smoking are around US$ 500 billion with a higher proportion in low and middle-income countries [4].

Tobacco is known as a global epidemic and a targeted, well-organized, and comprehensive approach is required to response this continually growing epidemic. The WHO Framework Convention on Tobacco Control (WHO FCTC) was launched in 2003 to response this demand and two years later in turned to an obligation that forces countries to implement couple of preventive and anti-tobacco activities and policies [5]. In 2008, WHO developed MPOWER package emphasizing on 6 most cost-effective tobacco control polices [6]. The results of previous studies showed that high level implementation of MPOWER package is associated with greater decrease in prevalence of tobacco smoking [7]. However, the status in Eastern Mediterranean Region is more sophisticated.

EMR is one the six geographic regions of WHO including 22 countries and nearly 679 million population. According to Atlas Tobacco, 14 out 22 of EMR countries grow tobacco and at least in seven countries tobacco is manufactured [8]. EMR is the only WHO region where smoking is expected to increase in men [9]. Moreover, there are serious concern regarding increase in proportion of tobacco smoker women over the recent future [9]. Tobacco is more affordable in EMR countries as they have the lowest average cigarette price [10]. In addition, other type of tobacco like water-pipe are a real concern in this region as they are too popular among women and youth [11]. There are also evidence regarding emergence and popularity of new tobacco products like electronic cigarette [12]. As it already mentioned the highest-level implementation of WHO FCTC articles could make a big progress in tobacco control on national scale. Despite the big progress in EMR countries, none of them have been able to implement these policies at the highest level and for many countries the pave is too long [9]. However, we believe that like any other program routine evaluation of tobacco control program in the EMR countries is necessary. In the current study, we aimed to evaluate the association between 10 years implementation of tobacco control policies including MPOWER package and cigarette affordability index and prevalence of tobacco in the EMR countries.

## Material and methods

### Tobacco control policies

We performed an ecologic study and countries in the EMRO region were our analysis units. As the study was performed on aggregate level data, we did not need any informed consent. Moreover, ethics approval for such research was waived by ethics committee and review board of Tehran University of Medical Sciences. In the current study, we used three sources of data including implementation of tobacco control policies on the MPOWER scale for years 2010, 2012, 2014, 2016, 2018, and 2020 [13], the tobacco affordability index (expressed as a percentage of GDP per capita required to purchase 2000 cigarettes for years 2010–2020, and age-standardized prevalence of tobacco smoking for each country in the EMRO region for years 2010, 2015, 2018, 2019, 2020, and 2023 [14]. MPOWER score was extracted for 16 countries in the EMRO region for the investigated years from WHO report on global tobacco epidemic. The MPOWER index assesses the implementation of anti-tobacco policies across seven key areas as the component W has two parts:

M: Monitoring tobacco use, P: Protecting people from tobacco smoke, O: Offering help to quit tobacco smoking, W: Warning about the dangers of tobacco and Anti-tobacco mass media campaign, E: Enforcing bans on tobacco advertising, R: Raising taxes on tobacco. Each of these areas contributes to the overall MPOWER score. The maximum score for the ’M’ component is 4, while for the remaining six components, it is 5. Consequently, the minimum attainable MPOWER score is 7, while the highest achievable score is 34 [15].

Cigarette affordability index was the other preventive measure that was investigated in the current study. To calculate cigarette affordability index, we extracted data on retail price for a pack of 20 cigarettes plus data on Gross domestic product (GDP) per capita over the investigated period for each EMR countries [16]. Data on cigarette price were extracted from the Global Health Observatory Data Repository. Moreover, for GDP per capita we used data from world bank [17]. Cigarette affordability index was described as proportion of the retail price for 100 packs of 20 cigarettes on Gross Domestic Product per capita for each EMR country [18]. Cigarette affordability index was calculated for the years 2010, 2012, 2014, 2016, 2018, and 2020.

### Tobacco prevalence

We obtained smoking prevalence for each country in the EMRO region from their national reports in 2010 and 2023 from Global Health Observatory Data Repository [14]. All types of tobacco including cigarettes, cigarillos, cigars, or water-pipe were regarded as tobacco, and the proportion of adults over 15 who answered yes for each of these products was considered as a current tobacco smoker. We removed six courtiers including Sudan, Somalia, Libya, Syria, Palestine, and Djibouti as there was no national estimates on tobacco smoking prevalence in 2010 or 2020.

### Statistical analysis

We summarized initial and final MPOWER score and tobacco prevalence for each country in the EMRO. Then we graphically described relative change in tobacco smoking prevalence from 2010 to 2023 by gender. The relative change of tobacco smoking prevalence was estimated through the following equation:

$$\left(\frac{{Prevalence}_{2023}-{Prevalnce}_{2010}}{{Prevalence}_{2010}}\right)*100$$ Eq 1

We used a fixed effect regression panel to investigate the association between MPOWER change score and change in prevalence of tobacco smoking over the past decade between 2010 and 2023. The association was calculated using the following equation

$$Prevalence_{it}=\beta_{1}+\beta_{1}MPOWER_{i,t}+Country_{i}+Year_{t}+\varepsilon_{it}$$ Eq 2

Where, Prevalence_it_ was age-standardized prevalence of tobacco smoking in country i at time t. MPOWER_i,t_ was MPOWER score for the country i at time t. Country and year were entered into the models as the fixed-effects. The country fixed-effect omitted variables that were constant over time and differe across the countries, while for time it was vise versa [15].

Then, we analyzed the association between change in cigarette affordability index and change in prevalence of tobacco smoking using the following equation:

$$Prevalence_{it}=\beta_{1}+\beta_{1}\;affordabilityindex_{i,t}+Country_{i}+Year_{t}+\varepsilon_{it}$$ Eq 3

In this equation affordabilityindex_i,t_ was the proportion of price 100 pack of the most sold cigarette brand (each pack contain 20 cigarettes) on GDP per capita in i country at time t. Then, to calcualte the simultaouns assocaition of MPOWER score and cigarette affordability index and prevalence of tobacco smoking the Eq 4 was used.


$$Prevalence_{it}=\beta_{0}+\beta_{1}\;MPOWER_{i,t}+\beta_{2}affordabilityindex_{i,t}+Country_{i}+Year_{t}+\varepsilon_{it}$$ Eq 4

We also used a multiple fixed-effect regression model to investigate the net association between change in each component of MPOWER index and change in prevalence of tobacco smoking in the EMRO. We repeated the analysis for men, women, and both sexes, separately. All statistical analysis was performed using Stata software and significance (Version 14.1, StataCrop LLC, College Station, TX, USA) level was P<0.05.

## Results

Table 1 describes the MPOWER score in 2010 and 2020 for each EMR country. Overall, the median MPOWER score for the EMRO region was 22.5 in 2010 that increased to 26.0 in 2020. The average tobacco smoking prevalence for the EMR countries was 18.2% in 2010 that downed to 15.9% in 2023. In 2010, the average affordability index for the EMRO stood at 2.3%, but by 2020, this index had increased to 6.2%. Tobacco smoking prevalence for men was 31.0% in 2010 and it reduced to 27.8% in 2023 (Table 1).

**Table 1:** MPOWER score and prevalence of tobacco smoking in 2010 and 2020.

	MPOWER		Tobacco prevalence						Affordability index (%)	
			Men		Women		Both sexes			
Country	2010	2020	2010	2023	2010	2023	2010	2023	2010	2020
**Afghanistan**	16	21	23.4	15.0	4.6	1.8	14.0	8.4	4.0	7.5
**Bahrain**	22	26	30.2	24.1	6.0	4.1	18.1	14.1	0.9	2.6
**Egypt**	31	30	41.3	41.8	0.6	0.3	21.0	21.1	2.8	3.1
**Iran**	27	28	23.4	17.0	2.4	1.0	12.9	9.0	0.7	15.6
**Iraq**	17	25	36.1	35.1	3.6	1.5	19.9	18.3	1.0	1.0
**Jordan**	27	29	53.7	57.5	11.4	13.4	32.6	35.5	3.4	7.8
**Kuwait**	23	26	34.6	33.0	3.1	2.0	18.9	17.5	0	1.1
**Lebanon**	23	24	41.4	42.3	28.2	25.1	34.8	33.7	1.9	3.0
**Morocco**	25	25	29.0	22.2	1.1	0.6	15.1	11.4	7.3	6.9
**Oman**	17	21	13.6	14.3	0.4	0.3	7.0	7.3	0.7	3.4
**Pakistan**	22	30	29.9	23.1	5.3	2.8	17.6	13.0	3.6	3.6
**Qatar**	20	29	19.3	18.4	2.2	1.5	10.8	10.0	0.2	1.2
**Saudi Arabia**	21	30	22.7	23.8	2.4	1.7	12.6	12.8	1.0	3.8
**Tunisia**	24	25	46.7	38.5	2.7	1.5	24.7	20.0	3.1	3.6
**UAE**	24	28	21.6	14.3	1.9	1.6	11.7	8.0	0.6	1.5
**Yemen**	21	24	29.8	25.4	9.1	6.0	19.5	15.7	6.0	34.2
**EMRO**	22.5	26	31.0	27.8	5.3	4.1	18.2	16.0	2.3	6.2

Tobacco smoking prevalence relative change in men ranged from -35.9% in Afghanistan to 7.1% in Jordan. Afghanistan, Iran, UAE, and Morocco had the highest reduction in tobacco smoking prevalence, whereas Jordan, Oman, Saudi Arabia, Lebanon, and Egypt showed an increase in the prevalence of tobacco smoking in men. We found a decreasing pattern in the prevalence of tobacco smoking in women in the EMR countries where tobacco prevalence decreased by 1.3% overall. The highest relative reduction was -60.9% that observed in Afghanistan. Iran (-58.3%), Iraq (-58.3%), Egypt (-50.0%), Pakistan (-47.2%), Morocco (-45.4%), Tunisia (-44.4%), Kuwait (-35.4%), and Yemen (-34.0%) were the other countries with the highest decrease in prevalence of tobacco smoking in women. On the other hand, Jordan showed substantial increase (17.7%) in women’s tobacco smoking prevalence (Table 2).

**Table 2:** Tobacco smoking prevalence relative change from 2010 to 2023 in the EMR countries stratified by gender.

	Tobacco smoking relative change (%)		
Country	Both	Men	Women
Afghanistan	-40.0%	-35.9%	-60.9%
Bahrain	-22.1%	-20.2%	-31.7%
Egypt	0.5%	1.2%	-50.0%
Iran	-30.2%	-27.4%	-58.3%
Iraq	-8.0%	-2.8%	-58.3%
Jordan	8.9%	7.1%	17.5%
Kuwait	-7.4%	-4.6%	-35.5%
Lebanon	-3.2%	2.2%	-11.0%
Morocco	-24.5%	-23.4%	-45.5%
Oman	4.3%	5.1%	-25.0%
Pakistan	-26.1%	-22.7%	-47.2%
Qatar	-7.4%	-4.7%	-31.8%
Saudi Arabia	1.6%	4.8%	-29.2%
Tunisia	-19.0%	-17.6%	-44.4%
UAE	-31.6%	-33.8%	-15.8%
Yemen	-19.5%	-14.8%	-34.1%
EMRO	-12.1%	-10.3%	-22.6%

We found an inverse association between MPOWER score improvement and tobacco smoking prevalence indicating that improvement in MPOWER score during the investigated period could lead to a significant reduction in the prevalence of tobacco smoking and observed association was statistically significant (β regression = -0.19, P-value = 0.001) (Table 2). The observed association was double in men (β regression = -0.26) compared with women (β regression = -0.12). According to the regression model, each unit increase in cigarette affordability index was associated with 0.9% reduction in the prevalence of tobacco smoking in EMR countries. The observed association was statistically significant (P-value = 0.001). Increasing MPOWER score and affordability index simultaneously was associated with 0.24% reduction in prevalence of tobacco smoking in the EMR countries and the observed association was statistically significant (P-value<0.001) (Table 3).

**Table 3:** Fixed-effects regression to assess the association between MPOWER change score, and 10 years change in prevalence of tobacco smoking in 16 countries in the EMR countries.

Coefficients	MPOWER		Affordability		MPOWER and affordability	
	β (95% CI)	P-value	β (95% CI)	P-value	β (95% CI)	P-value
Both gender	-0.19 (-0.28, -0.09)	0.001	-0.09 (-0.15, -0.02)	0.006	-0.24 (-0.39, -0.09)	<0.001
Male	-0.26 (-0.42, -0.10)	0.001	-0.12 (-0.22, -0.01)	0.022	-0.32 (-0.58, -0.06)	<0.05
Female	-0.12 (-0.17, -0.07)	0.001	-0.06 (-0.09, -0.02)	0.001	-0.16 (-0.24, -0.07)	<0.001

• Β = Regression coefficient, CI = Confidence interval

After adjustment for multiple confounders, we observed significantly inverse association monitoring tobacco (β = -0.41), health warning (β = -0.45), and tobacco smoking prevalence change. According to the Table 4, any improvement in implementation of these policies was associated with reduction in the prevalence of tobacco smoking in EMR countries and the observed difference was statistically significant (P-value<0.05). We repeated the analysis for each gender separately (Table 4).

**Table 4:** Multiple adjusted fixed-effect regression model analyzing the association between each MPOWER subdomain and the change in tobacco smoking prevalence across 16 EMR countries.

	Monitor	Protect people from tobacco smoke	Offer help to quite	Health warnings	Anti-Tobacco campaign	Advertising ban	Rising tax
	B (SE)	B (SE)	B (SE)	B (SE)	B (SE)	B (SE)	B (SE)
**Total**	-0.41 (0.19)	-0.31 (0.22)	0.23 (0.32)	-0.45 (0.21)	-0.10 (0.30)	-0.30 (0.21)	0.07 (0.21)
**P-value**	0.035	0.166	0.466	0.040	0.303	0.178	0.736
**Gender**							
**Male**	-0.58 (0.33)	-0.37 (0.39)	0.24 (0.55)	-0.68 (0.37)	-0.11 (0.17)	-0.19 (0.37)	-0.31 (0.36)
**P-value**	0.087	0.342	0.657	0.074	0.531	0.686	0.392
**Female**	-0.31 (0.09)	-0.34 (0.10)	0.07 (0.15)	-0.22 (0.10)	-0.02 (0.04)	-0.29 (0.10)	0.15 (0.01)
**P-value**	0.001	0.002	0.607	0.035	0.699	0.006	0.126

Β = Regression coefficient, SE = Standard Error, Each component was adjusted for all other components of MPOWER index.

## Discussion

The current study aimed to assess the relationship between changes in the implementation of tobacco control policies and the prevalence of tobacco smoking across EMR countries over the last decade. Additionally, we sought to explore the association between changes in cigarette affordability and the prevalence of tobacco smoking, considering it as a complementary preventive measure. Our analysis revealed promising trends, showing improvements in tobacco smoking prevalence across 12 out of the 16 investigated EMR countries. We observed an increase in the proportion of male tobacco smokers in Oman, Egypt, Saudi Arabia, Lebanon, and Jordan, while only Jordan experienced a similar trend among women. The study underscored the significance of enhancing MPOWER scores in contributing to the reduction of tobacco smoking prevalence in EMR countries over the past decade. Furthermore, we observed an upward trend indicating that cigarette became less affordable in the EMRO and there was an inverse relationship between the cigarette affordability index and the prevalence of tobacco smoking in these EMR countries. Moreover, our findings suggested that each unit improvement in the MPOWER score, and the affordability index additively was associated with a 0.26% reduction in tobacco smoking prevalence across the EMR countries during the investigate timeframe.

Our research revealed a statistically significant inverse correlation between the advancement in the implementation of anti-tobacco policies measured by the MPOWER scale over the study period and the changes observed in tobacco prevalence among both men and women. These findings align with previous studies, which have consistently reported a similar negative correlation [7, 19]. Studies conducted in European countries have also demonstrated a clear inverse relationship between the implementation of tobacco control policies and the prevalence of tobacco smoking [20]. Moreover, prior research has consistently indicated that the stringent application of tobacco control policies at their highest levels is associated with a reduction in tobacco prevalence [21]. However, it’s important to note that a study conducted by Husain et al. did not find a significant association between the MPOWER score and the prevalence of tobacco smoking on a global scale [15]. They have discussed that effect of tobacco preventive policies were strongly associated with initial status of the country regarding tobacco smoking prevalence and MPOWER status [15]. These policies were only effective in countries with initially high prevalence of tobacco smoking or high MPOWER score.

In our study, we specifically delved into the concept of cigarette affordability rather than solely focusing on cigarette price or tax percentages. This approach was motivated by the considerable divergence in economic development experienced by various countries within the EMRO over the past decade. On one hand, nations such as Saudi Arabia, Qatar, UAE, and Kuwait witnessed substantial increases in their GDP per capita. Conversely, countries like Iran, Syria Republic, Lebanon, Afghanistan, and Yemen encountered contrasting situations marked by economic challenges, declines in GDP per capita due to factors like international sanctions, civil conflicts, and political reforms [17]. Our study findings unveiled a trend across most EMR countries showcasing progress in terms of the cigarette affordability index. Over the past decade, efforts were successful in rendering cigarettes less affordable across the region [22]. However, this change varied significantly, ranging from a remarkable 28.2% increase in Yemen and 14.9% in Iran, marking it as the leading countries in this regard, to a marginal -0.4% change observed in Morocco. However, as previously highlighted, the substantial shifts in the affordability index of cigarettes within countries like Iran and Yemen were not solely a consequence of increased cigarette prices or taxation. These changes were also significantly influenced by economic crises leading to declines in GDP per capita. In numerous EMR countries, there remains significant room for improvement in this aspect, considering that the affordability index stands considerably lower than WHO-FCTC recommendation and developed countries [23].

Our study indicated a significant inverse association between cigarette affordability index and tobacco smoking prevalence indicating that the less affordability of cigarettes could decrease the prevalence of tobacco smoking over the study period. The robust correlation between cigarette affordability and smoking was previously highlighted by Blecher et al. in a global study [24]. Their research revealed that for every percent increase in the cigarette affordability index could decrease cigarette consumption by approximately 0.49–0.57% [24]. The disparity between these figures in our current study can be largely attributed to various factors, including the focus on tobacco prevalence in our research rather than cigarette smoking prevalence, differences in the time frames studied, and variations in the socioeconomic statuses of the countries involved. Similar findings was also reported in a study from ten southeastern European countries Increasing tobacco prices through tobacco taxation is already known as the most cost-effective approach to tobacco smoking cessation [25, 26]. Implementation of tobacco taxation at the highest level is pretty tough, however, even minor changes in tobacco prices in the low and middle-income countries may provide considerable outcomes due to the higher sensitivity of smokers to tobacco prices in such countries [21]. Guindon et al. have previously argued that individuals with lower socioeconomic status are more impacted by fluctuations in tobacco prices compared to those with higher socioeconomic status [27]. This discrepancy is attributed to the higher price elasticity of demand for cigarettes among the former group [23].

Furthermore, our analysis revealed that, even after adjusting for other facets within the MPOWER framework, the enhancement in implementing policies such as monitoring tobacco use and bolstering health warnings correlated with a reduction in tobacco smoking across EMR countries. These findings align with prior research indicating the effectiveness of health warnings on tobacco packaging as a preventive measure as it probably is the most cost-effective way of increasing public awareness regarding harmful effect of tobacco smoking [28, 29]. Such warnings not only deter non-smokers from initiating the habit but also serve as an incentive for smokers to consider quitting. The cost-effectiveness and feasibility of incorporating health warnings onto tobacco packages have made it a priority among low- and middle-income countries in their efforts toward tobacco prevention [28]. Notably, within the EMRO significant improvement in tobacco preventive measures have been observed, particularly in the realm of implementing impactful health warnings on tobacco products [30]. In the EMR countries, policies such as creating smoke-free public places and imposing bans on tobacco advertisements have been widely implemented [30]. However, a prevalent challenge undermining the effectiveness of these policies is the inadequate adherence and enforcement. Violations are frequently observed, significantly reducing the impact of these measures in terms of effectively controlling tobacco use within the region [31].

### Limitations and strengths

The current study was ecologic and therefore it is difficult to establish a causal association between the tobacco control policies and the change in prevalence of tobacco smoking. We had to exclude a couple of countries due to limited evidence regarding either MPOWER score or tobacco surveys and it reduced our statistical power and consequently limited us to generalize our findings to whole countries in the region. In addition, the data utilized in the current study, like any dataset or repository, may presented limitations concerning data quality, completeness, consistency, reporting discrepancies, and potential underreporting or misreporting. However, the current study was the first attempt to assess the impact of 10 years of tobacco control policies in the EMR countries with the best available data.

## Conclusion

The study’s findings suggest a successful association between enhanced implementation of tobacco control policies and a reduction in tobacco smoking prevalence among both men and women in EMR countries. Additionally, reducing the affordability of cigarettes could serve as a complementary preventive measure to further decrease tobacco smoking prevalence in the region.

## References

[1] Koronaiou K, Al-Lawati JA, Sayed M, Alwadey AM, Alalawi EF, Almutawaa K, et al. Economic cost of smoking and secondhand smoke exposure in the Gulf Cooperation Council countries. Tob Control 2021;30:680–6. doi: 10.1136/tobaccocontrol-2020-055715 32817575 PMC8543216

[2] GBD 2016 Risk Factors Collaborators. Global, regional, and national comparative risk assessment of 84 behavioural, environmental and occupational, and metabolic risks or clusters of risks, 1990–2016: a systematic analysis for the Global Burden of Disease Study 2016. Lancet 2017;390:1345–422. doi: 10.1016/S0140-6736(17)32366-8 28919119 PMC5614451

[3] Nemati S, Mohebbi E, Toorang F, Hadji M, Hosseini B, Saeedi E, et al. Population attributable proportion and number of cancer cases attributed to potentially modifiable risk factors in Iran in 2020. Int J Cancer 2023;153:1758–65. doi: 10.1002/ijc.34659 37548110

[4] Goodchild M, Nargis N, Tursan d’Espaignet E. Global economic cost of smoking-attributable diseases. Tob Control 2018;27:58–64. doi: 10.1136/tobaccocontrol-2016-053305 28138063 PMC5801657

[5] Yach D, Hawkes C, Epping-Jordan JE, Galbraith S. The World Health Organization’s Framework Convention on Tobacco Control: implications for global epidemics of food-related deaths and disease. J Public Health Policy 2003;24:274–90. 15015861

[6] MPOWER: a policy package to reverse the tobacco epidemic n.d. https://www.who.int/publications-detail-redirect/9789241596633 (accessed November 26, 2023).

[7] Dubray J, Schwartz R, Chaiton M, O’Connor S, Cohen JE. The effect of MPOWER on smoking prevalence. Tob Control 2015;24:540–2. doi: 10.1136/tobaccocontrol-2014-051834 25492934

[8] Eastern Mediterranean Region—TobaccoTactics n.d. https://tobaccotactics.org/article/eastern-mediterranean-region/ (accessed November 26, 2023).

[9] Al-Lawati JA, Mackay J. Tobacco control in the Eastern Mediterranean Region: the urgent requirement for action. East Mediterr Health J 2020;26:6–8. doi: 10.26719/2020.26.1.6 32043540

[10] Gordon MRP, Perucic A-M, Totanes RAP. Cigarette affordability in the Eastern Mediterranean Region. East Mediterr Health J 2020;26:55–60. doi: 10.26719/2020.26.1.55 32043546

[11] Aboaziza E, Eissenberg T. Waterpipe tobacco smoking: what is the evidence that it supports nicotine/tobacco dependence? Tob Control 2015;24 Suppl 1:i44–53. doi: 10.1136/tobaccocontrol-2014-051910 25492935 PMC4345797

[12] Al-Hamdani M, Brett Hopkins D. E-cigarettes in the Middle East: The known, unknown, and what needs to be known next. Prev Med Rep 2023;31:102089. doi: 10.1016/j.pmedr.2022.102089 36530454 PMC9747636

[13] GHO | By category | MPOWER Overview—Data by country n.d. https://apps.who.int/gho/data/node.main.TOBMPOWER?lang=en (accessed November 26, 2023).

[14] Age-standardized estimates of current tobacco use, tobacco smoking and cigarette smoking (Tobacco control: Monitor) n.d. https://www.who.int/data/gho/data/indicators/indicator-details/GHO/gho-tobacco-control-monitor-current-tobaccouse-tobaccosmoking-cigarrettesmoking-agestd-tobagestdcurr (accessed November 26, 2023).

[15] Husain MJ, Datta BK, Nargis N, Iglesias R, Perucic A-M, Ahluwalia IB, et al. Revisiting the association between worldwide implementation of the MPOWER package and smoking prevalence, 2008–2017. Tob Control 2021;30:630–7. doi: 10.1136/tobaccocontrol-2020-055758 32893187 PMC8543233

[16] GHO | By category | Retail price for a pack of 20 cigarettes—Data by country. WHO n.d. https://apps.who.int/gho/data/view.main.TOBRETAILv (accessed November 26, 2023).

[17] World Bank Open Data. World Bank Open Data n.d. https://data.worldbank.org (accessed November 26, 2023).

[18] Zubović J, Zdravković A, Jovanović O, Djukić M, Vladisavljević M. Affordability of cigarettes in ten Southeastern European countries between 2008 and 2019. Tob Control 2023:tc-2022-057716. doi: 10.1136/tc-2022-057716 37094936 PMC11187392

[19] Feliu A, Filippidis FT, Joossens L, Fong GT, Vardavas CI, Baena A, et al. Impact of tobacco control policies on smoking prevalence and quit ratios in 27 European Union countries from 2006 to 2014. Tob Control 2019;28:101–9. doi: 10.1136/tobaccocontrol-2017-054119 29472445 PMC6317447

[20] Gredner T, Mons U, Niedermaier T, Brenner H, Soerjomataram I. Impact of tobacco control policies implementation on future lung cancer incidence in Europe: An international, population-based modeling study. The Lancet Regional Health–Europe 2021;4. doi: 10.1016/j.lanepe.2021.100074 34029359 PMC8121752

[21] Gravely S, Giovino GA, Craig L, Commar A, D’Espaignet ET, Schotte K, et al. Implementation of key demand-reduction measures of the WHO Framework Convention on Tobacco Control and change in smoking prevalence in 126 countries: an association study. Lancet Public Health 2017;2:e166–74. doi: 10.1016/S2468-2667(17)30045-2 29253448

[22] Affordability—percentage of GDP per capita required to purchase 2000 cigarettes of the most sold brand (Tobacco control: Raise taxes) n.d. https://www.who.int/data/gho/data/indicators/indicator-details/GHO/gho-tobacco-control-raise-taxes-r-afford-gdp (accessed November 26, 2023).

[23] Van Walbeek C, Filby S. Analysis of Article 6 (tax and price measures to reduce the demand for tobacco products) of the WHO Framework Convention on Tobacco Control. Tob Control 2019;28:s97–103. doi: 10.1136/tobaccocontrol-2018-054462 30045973 PMC6589458

[24] Blecher EH, van Walbeek CP. An international analysis of cigarette affordability. Tob Control 2004;13:339–46. doi: 10.1136/tc.2003.006726 15564616 PMC1747952

[25] Ekpu VU, Brown AK. The Economic Impact of Smoking and of Reducing Smoking Prevalence: Review of Evidence. Tob Use Insights 2015;8:1–35. doi: 10.4137/TUI.S15628 26242225 PMC4502793

[26] Jha P, Peto R. Global effects of smoking, of quitting, and of taxing tobacco. N Engl J Med 2014;370:60–8. doi: 10.1056/NEJMra1308383 24382066

[27] Guindon G, Perucic A, Boisclair D. Higher Tobacco Prices and Taxes in South-East Asia An Effective Tool to Reduce Tobacco Use, Save Lives and Generate Revenue. Tobacco Control 2003.

[28] Monárrez-Espino J, Liu B, Greiner F, Bremberg S, Galanti R. Systematic review of the effect of pictorial warnings on cigarette packages in smoking behavior. Am J Public Health 2014;104:e11–30. doi: 10.2105/AJPH.2014.302129 25122019 PMC4167086

[29] Cunningham R. Tobacco package health warnings: a global success story. Tob Control 2022;31:272–83. doi: 10.1136/tobaccocontrol-2021-056560 35241600

[30] Heydari G, Zaatari G, Al-Lawati JA, El-Awa F, Fouad H. MPOWER, needs and challenges: trends in the implementation of the WHO FCTC in the Eastern Mediterranean Region. East Mediterr. Health J 2018;24:63–71. 29658622

[31] Saeedi E, Abdi S, Dardashti AR, Vand Rajabpour M. Tobacco control score scale in the Eastern Mediterranean countries: A comparative study between 2009 and 2021. East Mediterr Health J. 2023;29(12). 10.26719/emhj.23.120 38279865

